# CD4 expression on monocytes correlates with recovery from multiple organ dysfunction syndrome and mortality in patients with septic shock

**DOI:** 10.3389/fmed.2024.1328719

**Published:** 2024-05-10

**Authors:** Yingqian Sun, Yan Lu, Rui Xing, Yongjun Zhang, Longyi Zhang

**Affiliations:** ^1^Clinical Laboratory, Affiliated Dongyang Hospital of Wenzhou Medical University, Zhejiang, China; ^2^The Department of Hematology, Affiliated Dongyang Hospital of Wenzhou Medical University, Zhejiang, China

**Keywords:** septic shock, multiple organic dysfunction syndrome, CD4, logistic models, monocytes

## Abstract

**Background:**

To date, the correlation between CD4 on the monocytes (mCD4) expression and the prognosis of patients with septic shock remains unclear. The purpose of this study was to analyze the expression of mCD4 in these patients and further evaluate whether mCD4 expression correlates with either the recovery from multiple organ dysfunction syndrome (MODS) or mortality.

**Methods:**

The study participants were recruited from a tertiary general hospital in China (Affiliated Dongyang Hospital of Wenzhou Medical University). Sepsis and septic shock were diagnosed based on the diagnostic criteria of Sepsis-3. MODS was defined as a Sequential Organ Failure Assessment score of at least two organ systems ≥2. Persistent MODS was defined as the continual meeting of the MODS criteria when re-evaluated one week after admission (day 7). A logistic regression model was used to test whether mCD4 was an independent prognostic factor for mortality in patients with septic shock. A paired sample rank sum test was used to examine the correlation between mCD4 expression and MODS recovery.

**Result:**

The study recruited 79 patients with septic shock as the study group, 74 patients with sepsis as the disease control group, and 56 volunteers as healthy controls. In the first 24 h after admission (day 1), mCD4 expression was significantly reduced in patients with septic shock compared to healthy controls and patients with sepsis. Moreover, mCD4 expression was an independent prognostic factor for in-hospital and 28 day mortality in patients with septic shock. mCD4 expression did not show significant differences in patients with persistent MODS on day 7 compared to day 1. However, mCD4 expression was significantly higher in patients without persistent MODS on day 7 than on day 1.

**Conclusion:**

mCD4 expression is significantly reduced in patients with septic shock, which is an independent prognostic factor for mortality and closely related to recovery from MODS.

## 1 Introduction

Sepsis is the main cause of admission to intensive care units and is associated with a prolonged hospital stay, significantly increased mortality rates, and increased healthcare costs (1–3). Septic shock is a subtype of sepsis with a higher mortality risk that exhibits an uncontrollable inflammatory cascade and severe immune suppression and is often accompanied by multiple organ failure syndrome (MODS) (4, 5).

Representing innate immunity, monocytes can inhibit adaptive immune responses by reducing antigen presentation. In previous studies, the role of Human Leukocyte Antigen (HLA)-DR on the surface of monocytes (mHLA-DR) in coordinating the local immune response of monocytes in patients with sepsis has been demonstrated (6). The expression of mHLA-DR in patients with sepsis is reduced, and this is related to the patients’ prognoses (7–9). CD4 on the surface of monocytes (mCD4) is an independent signal transduction receptor (10) that is closely related to the evolution of monocytes into mature macrophages (11). The expression of mCD4, like that of mHLA-DR, is also influenced by immune regulation (12).

Currently, the correlation between mCD4 expression and the prognosis of patients with septic shock remains unclear. In addition, previous studies have shown that patients with accompanying MODS status during sepsis diagnosis experience a longer treatment recovery process than patients with septic shock but without MODS do (13). Moreover, long-term dysfunction can lead to irreversible organ failure. Therefore, an investigation of patient recovery is urgently required to guide physicians in providing timely intervention for patients with potentially poor prognoses. Therefore, the purpose of this study was to analyze the expression level of mCD4 in patients with septic shock and further evaluate its correlation with both the recovery of MODS and mortality.

## 2 Materials and methods

### 2.1 Study population

Patients with septic shock were consecutively recruited from a tertiary general hospital (Dongyang Hospital Affiliated with Wenzhou Medical University) in China from March 2021 to September 2023 and included in the study group. Additionally, those with sepsis were consecutively recruited from March 2023 to September 2023 and included in the disease control group. All patients were admitted to the emergency department or intensive care unit. The inclusion criteria for this study were: (1) age ≥ 18 years and (2) sepsis and septic shock diagnosis based on the diagnostic criteria of Sepsis-3 (14). The exclusion criteria were: (1) patients with known malignant tumors and autoimmune diseases; (2) pregnant or lactating patients; (3) patients with human immunodeficiency and chronic hepatitis C virus infections; and (4) patients with missing clinical data. Patients with MODS were defined as patients whose Sequential Organ Failure Assessment (SOFA) score for at least two organ systems was ≥2 within the first 24 h of admission (day 1). In addition, we recruited 56 healthy volunteers who were matched by age and sex but who had no major surgical history and did not suffer from any inflammatory diseases, malignant tumors, hematological diseases, or autoimmune diseases.

This study was approved by the Ethics Committee of the Affiliated Dongyang Hospital of Wenzhou Medical University (Approval #: 2021-YX-143). All participants or their guardians signed informed consent forms.

### 2.2 Data collection and sample testing

Patient demographic data were collected on day 1. The severity score was also evaluated on day 1. If a patient experiencing septic shock with MODS stayed in the hospital for more than 7 days, the patient was re-evaluated for severity 1 week after admission (day 7). The severity scores included the Acute Physiology and Chronic Health Evaluation (APACHE) II and SOFA scores.

For the procalcitonin (PCT) test, 2 mL of venous blood was collected in heparin lithium anticoagulant tubes. Plasma was collected after centrifugation and tested with an electrochemiluminescence immunoanalyzer (Cobas e601, Roche, Switzerland). C-reactive protein (CRP) was detected by scattering turbidimetry on a protein analyzer (PA-990 pro, Lifetronic, China).

For mCD4 detection, 2 mL of venous blood was collected from patients in EDTA anticoagulant tubes, and the mean fluorescence intensity of mCD4 was detected by flow cytometry within 24 h. The antibody combinations used for surface staining are listed in Supplementary Table S1. The staining process is described in Supplementary Figure S1, and the gating strategy is illustrated in Supplementary Figure S2.

### 2.3 Outcomes

The primary outcome of this study was the in-hospital and 28 day all-cause mortality of patients experiencing septic shock. The secondary outcome was improvement in organ failure from day 1 to day 7 in patients with septic shock in the portion of the population with MODS. Persistent MODS was defined as meeting the MODS criteria when re-evaluated on day 7.

Patients were followed up until death or October 30, 2023 (to ensure a follow-up of ≥28 days), whichever came first.

### 2.4 Statistical analysis

R software (version 4.1.0) was used for data analysis. Continuous variables were expressed as means ± standard deviations or medians [quartiles]. Categorical variables were expressed in terms of quantity and proportion. Comparisons between independent sample groups were conducted using the Mann–Whitney U test, and paired analysis of related samples was conducted using the paired sample rank sum test. The logistic regression model was used to test whether mCD4 was an independent risk factor for in-hospital and 28 day mortality in patients with septic shock. The results are represented by the odds ratio (OR) and the 95% confidence interval (95% CI). Multicollinearity between variables was determined based on the variance inflation factor (VIF). If the VIF was less than 10, it indicated that there was no multicollinearity between variables. Statistical significance was considered when *p* < 0.05.

## 3 Results

As shown in Supplementary Figure S3, 79 patients with septic shock and 74 patients with sepsis were finally included in this study. Without fungal detection, the positive rate of blood culture in patients with septic shock was 48.1%. The in-hospital mortality rate of patients with septic shock was 21.5% (*n* = 17), and the 28 day mortality rate was 25.3% (*n* = 20). In addition, 56 volunteers were recruited as healthy controls. The baseline characteristics of all study participants are presented in Table 1.

**Table 1:** Baseline characteristics of the study participants.

	Healthy control (*n* = 56)	Sepsis (*n* = 74)	Septic shock (*n* = 79)
Age, years	72 ± 11	73 ± 14	70 ± 15
Sex, *n* (%)			
Male	32 (57.1)	43 (58.1)	47 (59.5)
Female	24 (42.9)	31 (41.9)	32 (40.5)
Comorbidities, *n* (%)			
Diabetes		22 (29.7)	19 (24.1)
Hypertension		46 (62.2)	37 (46.8)
Coronary atherosclerotic heart disease		14 (18.9)	13 (16.5)
Positive blood cultures, *n* (%)		33 (44.6)	38 (48.1)
Pathogen type, *n* (%)			
Gram-positive bacteria		28 (37.8)	28 (35.4)
Gram-negative bacteria		10 (13.5)	6 (7.6)
Fungus		0 (0)	1 (1.3)
Anaerobic bacteriaceae		0 (0)	1 (1.3)
Mixed infection		5 (6.8)	12 (15.2)
Unknown		31 (41.9)	31 (39.2)
Severity scores			
SOFA		5 [3–8]	11 [8–15]
APACHE II		8 [4–11]	16 [11–23]
CRP (mg/L)		86.0 [19.8–153.2]	154.7 [77.4–187.1]
PCT (ng/mL)		4.1 [0.6–11.6]	32.5 [7.3–76.1]
In-hospital mortality, *n* (%)		1 (1.4)	17 (21.5)
28 day mortality, *n* (%)		2 (2.7)	20 (25.3)

APACHE, acute physiology and chronic health evaluation; CRP, C-reactive protein; PCT, procalcitonin; SOFA, sequential organ failure assessment.

### 3.1 mCD4 expression on day 1 and mortality in patients with septic shock

**Figure 1:**
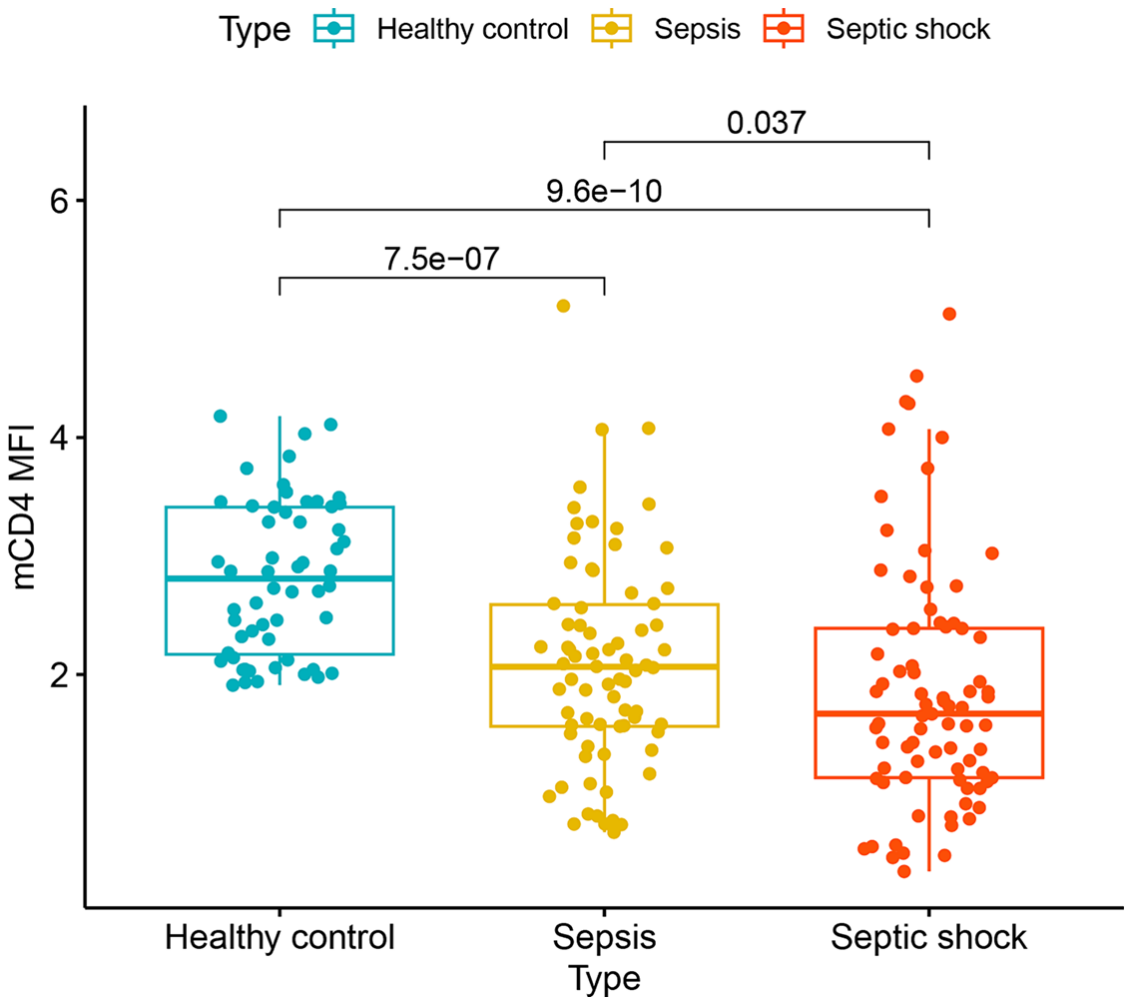
The expression of CD4 on monocytes in healthy controls, patients with sepsis, and patients with septic shock. mCD4, CD4 on the surface of monocytes; MFI, mean fluorescence intensity.

mCD4 expression was significantly reduced in patients experiencing septic shock compared to that of patients with sepsis and patients with healthy controls (OR [95% CI]: 1.67 [1.13–2.39] vs. 2.07 [1.56–2.60] vs. 2.81 [2.16–3.42]) (Figure 1).

In Table 2, a multivariate logistic regression was used to test whether mCD4 expression was an independent predictor of in-hospital and 28 day mortality in patients with septic shock. The covariates involved in the adjustment included age, sex, SOFA score, APACHE II score, CRP, PCT, and white blood cell. According to VIF results, there was no multicollinearity between variables. The results showed that mCD4 expression was an independent prognostic factor for in-hospital and 28 day mortality in patients with septic shock (OR [95% CI]: 0.17 [0.04–0.70]; 0.39 [0.16–0.97]).

**Table 2:** Multivariate logistic analysis of mortality in patients with septic shock.

Variables	In-hospital mortality			28 day mortality			VIF
	Odds ratio	95% Confidence interval	*p*-value	Odds ratio	95% Confidence interval	*p*-value	
mCD4	0.17	0.04–0.70	0.014	0.39	0.16–0.97	0.043	1.097
Age	1.13	1.03–1.24	0.011	1.09	1.02–1.17	0.009	1.161
Sex	4.32	0.87–21.4	0.073	2.23	0.60–8.28	0.232	1.051
SOFA	1.02	0.89–1.17	0.731	1.03	0.91–1.16	0.665	1.123
APACHE II	1.07	0.97–1.19	0.176	1.05	0.96–1.15	0.304	1.167
WBC	1.00	0.91–1.10	0.973	0.99	0.92–1.07	0.797	1.241
CRP	1.00	0.99–1.01	0.759	1.00	0.99–1.01	0.953	1.428
PCT	0.99	0.97–1.01	0.224	0.99	0.97–1.01	0.236	1.176

APACHE, acute physiology and chronic health evaluation; CRP, C-reactive protein; mCD4, CD4 on the surface of monocytes; PCT, procalcitonin; WBC, white blood cell; SOFA, sequential organ failure assessment.

### 3.2 mCD4 expression and recovery from MODS in patients with septic shock

Among the 79 patients with septic shock, 63 (79.7%) had MODS. After excluding patients who withdrew from the study due to death, transfer, or other personal reasons, 37 patients underwent continuous mCD4 testing on day 7. Among these patients, 19 had persistent MODS on day 7, while 18 without persistent MODS had recovered organ function either completely or partially.

**Figure 2:**
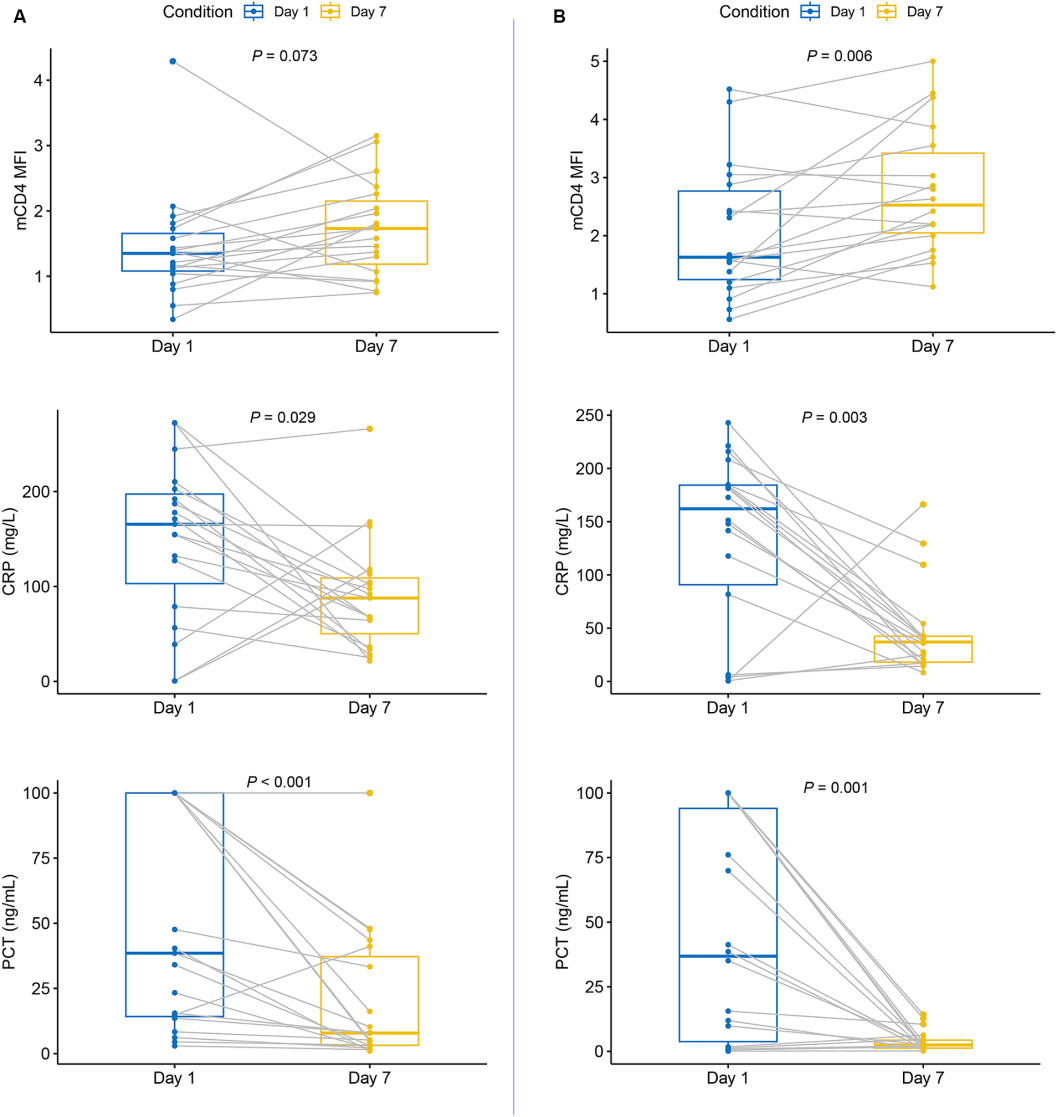
The expression of CD4 on monocytes, CRP levels, and PCT levels in patients experiencing septic shock with MODS on day 7. **(A)** Patients with persistent MODS on day 7; **(B)** patients without persistent MODS on day 7.

Patients with persistent MODS on day 7 exhibited no significant difference in mCD4 expression on day 1, whereas their CRP and PCT levels were significantly decreased (Figure 2A). Conversely, mCD4 expression was significantly increased in patients without persistent MODS on day 7 compared to that on day 1, whereas CRP and PCT levels were significantly decreased (Figure 2B).

## 4 Discussion

Patients admitted for septic shock have rapid disease progression and complex etiology and pathogenesis. Consequently, they are prone to delayed diagnosis and treatment, leading to increased patient mortality. Therefore, both early and efficient diagnosis and assessment of their condition and timely initiation of individualized treatment are crucial for improving the prognosis of patients with septic shock. In this study, a significant decrease in mCD4 expression was observed in patients with septic shock on admission, which was an early independent predictor of in-hospital and 28 day mortality. Moreover, the expression of mCD4 over time was closely related to organ function recovery.

CD4 is a membrane glycoprotein expressed on the surface of human monocytes; however, the correlation between its expression and monocyte function is unclear. Monocytes may be induced to differentiate into macrophages by activating CD4 (11). Concern regarding mCD4 expression in patients with human immunodeficiency virus was first reported in a correlation study (15, 16). Other studies have reported that mCD4 expression is significantly reduced in patients with severe coronavirus disease 2019 (COVID-19) (17, 18). In this study, mCD4 was significantly underexpressed in patients with septic shock. However, the mechanism of down-regulation of mCD4 expression triggered by septic shock needs to be elucidated in future studies.

For patients with septic shock, immune paralysis caused by an uncontrolled inflammatory cascade may explain their poor prognosis (19). Many inflammation-related biomarkers have been proven to be independently associated with the prognosis of patients with septic shock, including mid-regional pro-adrenomedullin (20), interleukin 6 (21), CD64 (22), and monocyte HLA-DR expression (23). CRP and PCT, the most commonly used clinical inflammatory markers, mainly reflect the pro-inflammatory host immune response (24, 25), and are widely used in the diagnosis of sepsis, but their prognostic value is limited (26, 27). This study proved that mCD4 was an independent predictor of the prognosis of patients with septic shock. Moreover, in patients with septic shock with MODS, changes in CRP and PCT were not related to organ function recovery, while changes in mCD4 levels were closely related. Therefore, mCD4 may provide more clinical prognostic information than CRP and PCT.

This study had some limitations. As this was a single-center study, further prospective multicenter studies are necessary to verify our findings. In addition, misclassification bias may have occurred due to the limited information on baseline medical conditions and organ function before admission, even though real-world practice was simulated.

## 5 Conclusion

mCD4 expression is significantly reduced in patients experiencing septic shock, which is an independent prognostic factor for mortality and closely related to recovery from MODS.

## Data availability statement

The original contributions presented in the study are included in the article/Supplementary material, further inquiries can be directed to the corresponding author.

## Ethics statement

The studies involving humans were approved by the Ethics Committee of the Affiliated Dongyang Hospital of Wenzhou Medical University. The studies were conducted in accordance with the local legislation and institutional requirements. The participants provided their written informed consent to participate in this study.

## Author contributions

YS: Writing – original draft, Conceptualization, Project administration, Validation, Writing – review & editing. YL: Data curation, Visualization, Writing – original draft. RX: Supervision, Visualization, Writing – review & editing. YZ: Formal analysis, Supervision, Writing – original draft. LZ: Project administration, Resources, Validation, Visualization, Writing – original draft, Writing – review & editing.
