# Supplementary material for: CD4 expression on monocytes correlates with recovery from multiple organ dysfunction syndrome and mortality in patients with septic shock
Source: Front Med (Lausanne). 2024 May 10;11:1328719. doi: 10.3389/fmed.2024.1328719 (PMC11116609; doi:10.3389/fmed.2024.1328719)
Supplement: Supplementary file 1 [file Data_Sheet_1.PDF]

CD4 expression on monocytes correlates with recovery from multiple organ dysfunction syndrome and mortality in patients with septic shock

Yingqian Sun[1†], Yan Lu[1†], Rui Xing[2], Yongjun Zhang[1], Longyi Zhang[1]*

[†]These authors contributed equally to this work

[1]Clinical Laboratory, Affiliated Dongyang Hospital of Wenzhou Medical University, Zhejiang, China

[2]The Department of Hematology, Affiliated Dongyang Hospital of Wenzhou Medical University, Zhejiang, China

*** Correspondence:**
Longyi Zhang
happy_zhang1y@163.com

Supplementary Table 1 Staining panel for monoclonal fluorescent antibody.

Fluorochrome	Marker	Clone	Source
FITC	CD20	clone B9E9	Beckman Coulter
FITC	CD3	clone UCHT1	Beckman Coulter
FITC	CD19	clone J4.119	Beckman Coulter
PE	CD123	clone SSDCLY107D2	Beckman Coulter
APC	CD14	clone RMO52	Beckman Coulter
AA700	CD56	clone N901	Beckman Coulter
AA750	CD4	clone 13B8.2	Beckman Coulter
KrO	CD45	clone J.33	Beckman Coulter

FITC, Fluorescein Isothiocyanate; PE, Phycoerythrin; ECD, Phycoerythrin-Texas; PE-Cy5.5, Phycoerythrin-Cyanin 5.5; APC, Allophycocyanin; AA700, APC-Alexa Fluor 700; AA750, APC- Alexa Fluor 750; KrO, Krome Orange

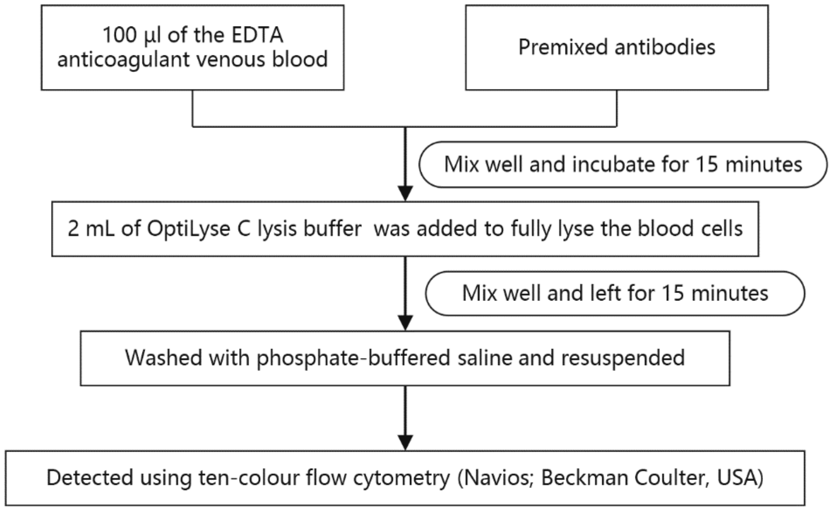

Supplementary Figure 1 The staining process of CD4 expression on monocyte detected by flow cytometry.

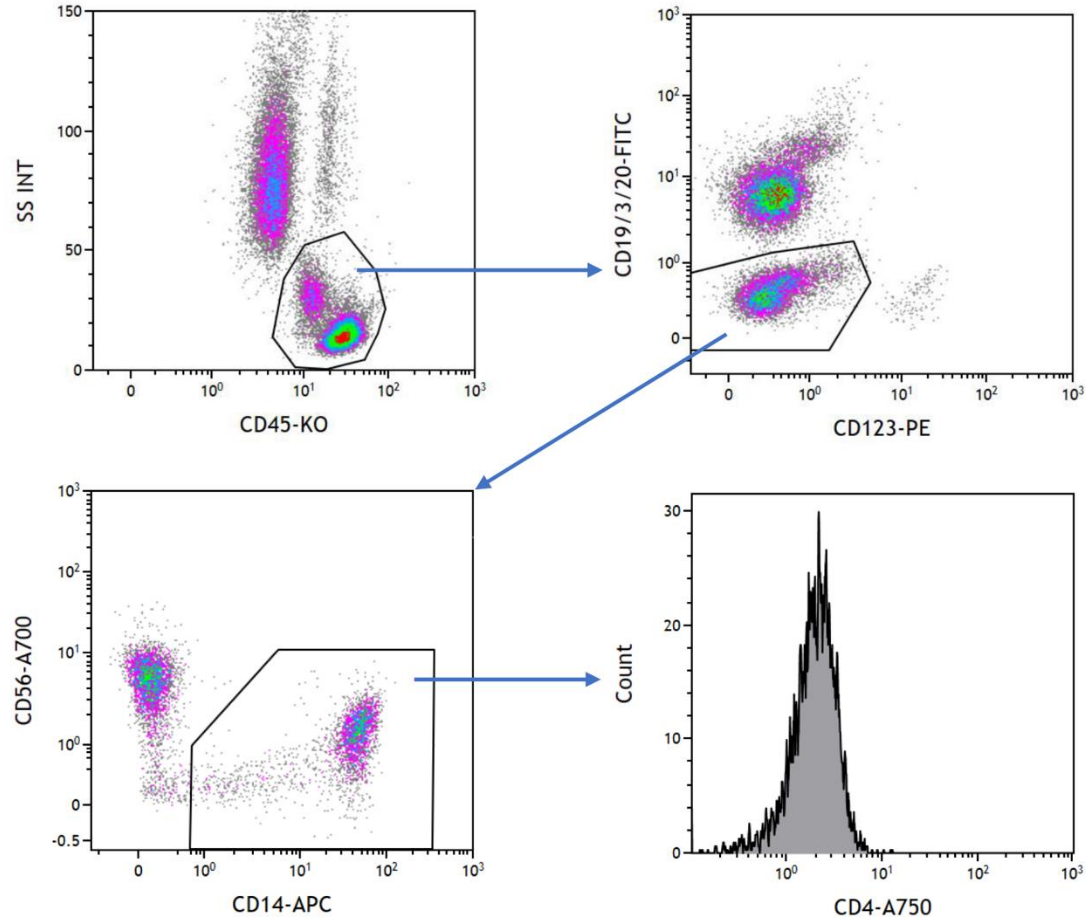

Supplementary Figure 2 The gating strategy of CD4 expression analysis on monocytes by flow cytometry

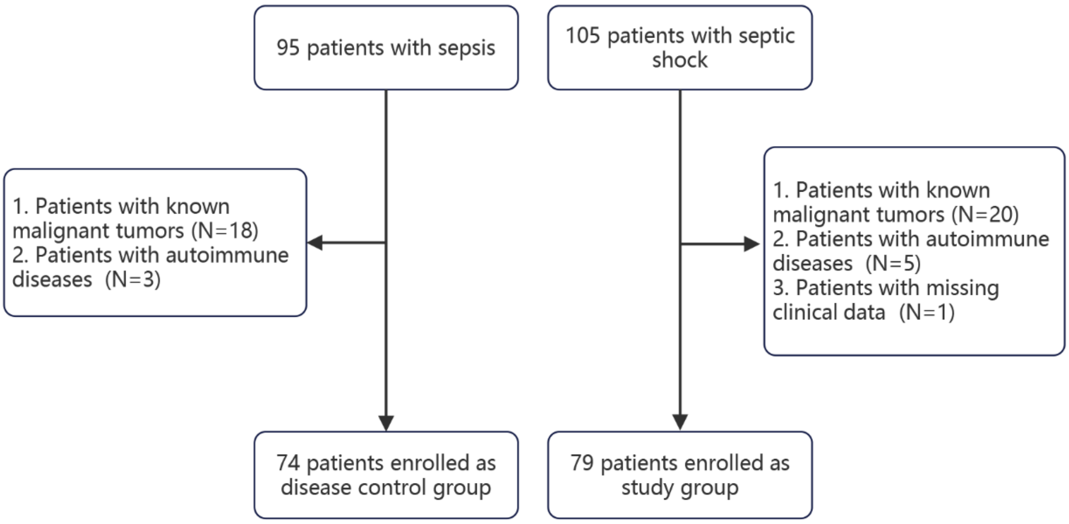

Supplementary Figure 3 Screening process of the disease control group and study group.
